# Analysis of Volatile Components of Cape Gooseberry (*Physalis peruviana* L.) Grown in Turkey by HS-SPME and GC-MS

**DOI:** 10.1155/2014/796097

**Published:** 2014-03-06

**Authors:** Murat Yilmaztekin

**Affiliations:** Department of Food Engineering, Faculty of Engineering, Inonu University, 44280 Malatya, Turkey

## Abstract

Volatile components in cape gooseberry fruit at ripe stage were collected using headspace-solid phase microextraction, and analyzed by gas chromatography-mass spectrometry. Three solid phase microextraction fiber coatings (DVB/CAR/PDMS, CAR/PDMS, and PDMS/DVB) were tested for evaluation of volatile compounds. DVB/CAR/PDMS fiber showed a strong extraction capacity for volatile compounds and produced the best result in case of total peak areas. A total of 133 volatile compounds were identified in fruit pulp; among them 1-hexanol (6.86%), eucalyptol (6.66%), ethyl butanoate (6.47%), ethyl octanoate (4.01%), ethyl decanoate (3.39%), 4-terpineol (3.27%), and 2-methyl-1-butanol (3.10%) were the major components in the sample extracts.

## 1. Introduction

Cape gooseberry (*Physalis peruviana* L.) is a cherry-sized, yellow-fleshed intriguing berry which was originally cultivated in the Andes. The round orange fruit is loosely enclosed in a papery husk which provides a natural wrapper for storing the fruit, as long as it is kept dry. Various species of the fruit have been subject to much confusion in the literature and in the trade.* Physalis peruviana* L. species which bears a superior fruit and has become widely known is commercially produced in Ecuador, South Africa, Kenya, Zimbabwe, Australia, New Zealand, Hawaii, India, Malaysia, Colombia, and China. Currently, the production of Cape gooseberry has expanded to tropical and subtropical countries such as the Caribbean and Colombia, the major producer [1]. In addition to being canned whole and preserved as jam, the Cape gooseberry is made into sauce, used in pies, puddings, chutneys, and ice cream, and eaten fresh in fruit salads and fruit cocktails. In Colombia, the fruits are stewed with honey and eaten as dessert [2, 3].

Aroma and flavour are one of the most important attributes and quality criteria that affect the consumption of fruits, and both qualitative and quantitative information is desired for characterizing aroma producing compounds [4]. The study of flavour on a more analytical and scientific basis has been achieved by the development and application of modern analytical techniques. Solvent extraction is one of the most used techniques for the volatile compound isolation; however, it is generally accomplished at high temperatures or under reduced pressure, conditions that can destroy or alter some volatile flavor compounds and/or produce artifacts [5]. Recently, for the purposes of determining fruit aromas, the solid-phase microextraction (SPME) technique has been applied as an alternative sample preparation strategy, to overcome the problems associated with conventional sampling methodologies. In addition, the SPME procedure more closely reflects the true flavor profile of the fruits than those that might be generated by solvent extraction processes [6].

Despite its importance, there are only few works regarding volatile composition [7, 8] and a natural precursor of cinnamic acid derived volatiles [9] of Cape gooseberry in the literature. To the best of my knowledge the flavouring components of Cape gooseberry fruit have not been determined by HS-SPME. The objective of this study was to analyze the volatiles of the fruit by HS-SPME extraction and to obtain more information about its flavour profile.

## 2. Materials and Methods

### 2.1. Fruit Samples and Chemicals

Cape gooseberry fruits were hand harvested at full ripe stage from plants growing in Antalya, Turkey, in September 2010. The berries were individually quick frozen (IQF) and stored at −18°C until analysis. Compounds used as references were purchased from Sigma-Aldrich-Fluka (Milan, Italy), Merck (Darmstadt, Germany), and Extrasynthese (Genay, France). A C_8_–C_20_ + C_20_–C_40_ *n*-alkane mixture, used for determination of Kovats' retention indices, was obtained from Sigma-Aldrich (St. Louis, MO, USA).

### 2.2. Isolation of Volatile Components

A method adapted from Pellati et al. [10] was used for HS-SPME. Three SPME fiber coatings, DVB/CAR/PDMS, CAR/PDMS, and PDMS-DVB, were screened to find the best coating for evaluation volatile compounds. A manual SPME device and fibers were obtained from the Supelco Company (Bellefonte, PA). Fibers were thermally conditioned in accordance with the manufacturer's recommendations before first use. 10 g of IQF fruits were thawed in a refrigerator (1°C). The sample was then pureed in a blender (turbo blender, Moulinex). A 0.5 g amount of cape gooseberry puree was hermetically sealed in a 15 mL screw top amber vial with a polypropylene hole cap and PTFE/silicone septa (Supelco, Bellefonte, PA, USA) and left to equilibrate 15 min in a temperature-controlled six-vial agitator tray at 40°C. Then, the SPME device was inserted into the sealed vial by manually penetrating the septum and the fiber was exposed to the fruit material headspace during the extraction time of 30 min. Following the sampling procedure, the SPME fiber was immediately inserted into the gas chromatograph (GC) splitless injection port (equipped with a 0.75 mm i.d. inlet liner) and thermally desorbed for 3 min at 250°C. Before each sampling procedure, fiber was reconditioned for 10 min in the injection port of gas chromatograph at 250°C. This reconditioning procedure was enough to guarantee no peaks in blank runs, and it was a good compromise between the chromatography runs and the extraction procedures. The extraction procedure was repeated three times for each type of fiber.

### 2.3. Gas Chromatography-Mass Spectrometry (GC-MS) Analyses

All analyses were performed on a Shimadzu 2010 (Shimadzu, Kyoto, Japan) GC equipped with a MS-QP 2010 (Shimadzu, Kyoto, Japan) series mass selective detector. The GC was fitted with a TRB-Wax (Teknokroma, Barcelona, Spain) fused silica capillary column (60 m × 0.25 mm i.d. and 0.25 *μ*m film thickness). Helium was used as the carrier gas at a flow rate of 1 mL/min. The column was maintained at 40°C for 5 min after injection then programmed at 3°C/min to 240°C, which was maintained for 15 min. The total run time including oven cooling was 86 min. Injector, transfer line temperature, and ion-source temperatures were 250°C. All mass spectra were acquired in electron-impact (EI) mode; the ionization voltage was 70 eV; the mass range was 35–450 m/z; scanning rate was 1 scan/s. A mixture of n-alkanes (C_8_–C_20_ + C_20_–C_40_) was injected under the above temperature program to calculate the retention indexes (as Kovats' indexes,* I*) of each compound.

The peaks were identified by comparison of the obtained mass spectra of the relevant chromatographic peaks with spectra of the NIST (National Institute of Standards and Technology, Gaithersburg, MD, USA) and Wiley libraries. In addition, the compounds were tentatively identified by comparing the experimental retention indices with the theoretical ones, which were obtained from the NIST Standard Reference Database [11]. Peak enrichment on coinjection with authentic reference compounds was also carried out. The comparison of the MS fragmentation pattern with those of pure compounds and mass spectrum database search was performed using the MS spectral databases.

## 3. Results and Discussion

### 3.1. Screening of Fibers

The extraction technique should be selected relative to the nature of sample matrix. In general, direct SPME is more sensitive than headspace SPME for analytes predominantly present in liquid. However, headspace SPME exhibits lower background than direct SPME, and is suitable for the extraction or more volatile analytes in most gaseous, liquid, and solid samples compounds [4]. Furthermore, headspace SPME sampling avoids interferences from nonvolatile matrix components and increases fiber life time [12]. On these grounds, headspace sampling technique was carried out to extract volatile compounds in this study.

**Figure 1:**
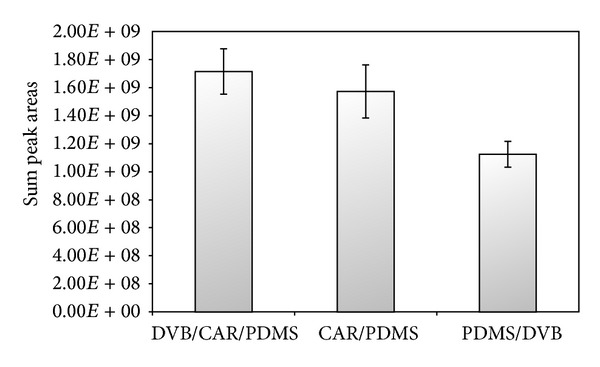
Comparison of different fiber coatings according to total peak area.

Three SPME fiber coatings, DVB/CAR/PDMS, CAR/PDMS, and PDMS-DVB, were selected for evaluation of volatile compounds. A response based on the sum of the peak areas is one of the most frequently used parameters to optimize the SPME extraction conditions or to select a fiber coating [10].  Figure 1 shows the comparison of different fiber coatings. The results of the fiber screening confirmed that the DVB/CAR/PDMS fiber showed a strong extraction capacity for volatile compounds and produced the best result in case of total peak areas. Given the better profile shown by this coating, this fiber was selected for characterization of the volatile compounds of Cape gooseberry. Bicchi et al. [13] observed that the most effective fibres for HS-SPME were those characterized by two components: a liquid (PDMS) for the less polar compounds and a solid (DVB, CAR or both) polymeric coating for the more polar constituents.

### 3.2. Volatile Compounds Identified in Cape Gooseberry

**Figure 2:**
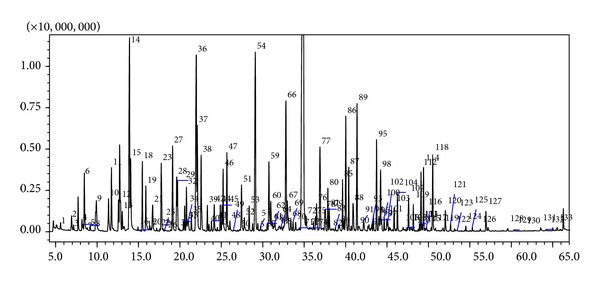
Total ion current (TIC) chromatogram of the HS-SPME volatile compounds of Cape gooseberry.

Figure 2 shows the total ion current (TIC) chromatogram of the HS-SPME volatile compounds of Cape gooseberry. The peak numbers in the chromatogram corresponded to the tentatively identified compounds listed in Table 1. In all, 133 different volatile compounds were identified and grouped in classes of substances, including 42 alcohols, 36 esters, 17 terpenes and derivatives, 13 aldehydes, 10 ketones, 4 lactones, 6 acids, and 5 oxides. In addition, terpene-derived aroma volatiles were grouped into several classes, for example, *β*-cyclocitral and geranial in aldehydes, linalool and geraniol in alcohols, and *β*-ionone and geranyl acetone in ketones.

#### 3.2.1. Alcohols

Alcohols were the most abundant volatile constituents which accounted for the largest proportion of the total volatiles (39.27%). 1-hexanol, followed by eucalyptol, and 4-terpineol were the alcohols found in highest concentration. The origin of C_6_ alcohols which is reported as an important contributor to the aroma of fresh (green and herbaceous notes), such as 1-hexanol, is related to the lipoxygenase activity [14]. This enzyme that occurs in plants, and, namely, in fruits, catalysis the oxidation of unsaturated fatty acids [15], as a first step to the production of compounds such as the short chain alcohols. There are a group of terpenoid flavour volatile compounds which possess strong effects on the human appreciation. This very diverse group of compounds is presumably generated by an oxidative cleavage of the carotenoid (tetraterpenoids, C_40_) molecule between the C_9_ and C_10_ positions, yielding apocarotenoids (also called norisoprenes) with 13 carbon atoms. Although other apocarotenoids of 9–20 carbon atoms are present in nature, only C_13_ has been described to have an important role in some fruit flavours and in the scent of some flowers [16, 17].

#### 3.2.2. Esters

The next most abundant compounds were esters comprising 38.52% of the total volatile components identified. Among these esters, ethyl butanoate, ethyl octanoate, and ethyl decanoate were the esters found in greatest concentration. Aliphatic esters contribute to the aroma of nearly all fruits and many other foods. Some are also responsible for the smell of a particular flower; however, many of these esters possess a nonspecific fruity odor.

As the number of carbon atoms increases, the odor changes to fatty soapy and even metallic. The straight-chain ester constituents are believed to be synthesized via *β*-oxidation of fatty acid, which may be then reduced to the corresponding alcohols before transesterification [18]. Alcohol acyltransferases are responsible for the transfer of alcohol to acyl-CoA, resulting in the synthesis of a wide range of esters [15, 19, 20].

#### 3.2.3. Terpenes and Derivatives

Terpenes and derivatives are the next more abundant compounds comprising 7.31% of the volatile components determined. Among these, *α*-terpinolene, *β*-myrcene, and cymenene were detected at the highest levels. All terpenoids are derived by repetitive fusion of branched five carbon units based on isopentane skeleton. Many of them are volatile, as hemiterpenes (C_5_), monoterpenes (C_10_), sesquiterpenes (C_15_), and even some diterpenes (C_20_). Terpenes are derived either from mevalonate pathway, which is active in cytosol and starts from acetyl-CoA, or from methylerythritol-4-phosphate pathway, which is active in the plastids and starts from pyruvate and glyceraldehyde-3-phosphate [21]. On the other hand, the biosynthesis of some terpene derived compounds can be explained by catabolic pathways in fruits. These are primarily oxidative degradation products of the carotenoids. Carotenoid oxidation occurs when the plant tissue is damaged or during ripening [22]. Terpenes and their derivatives have been identified at varying levels in most of the soft fruits [23], and they are responsible for the varietal character of the fruits being present, at least in part, as glycosides [15]. They were reported as volatile components responsible for a wide spectrum of aromas (woody, piney, turpentine-like, herbaceous, and terpy), mostly perceived as very pleasant [14, 15].

#### 3.2.4. Aldehydes

Aldehydes represent 7.05% of the total volatile compounds. Benzaldehyde, hexanal, and *β*-cyclocitral were the predominant aldehydes. In general, aldehydes are common in fruit flavours and are believed to play an important role in many fruits [24]. Fatty acids and amino acids are precursors of a great number of volatile aldehydes. Linoleic and linolenic acids in fruits and vegetables are subjected to oxidative degradation by lipoxygenase alone or in combination with a hydroperoxide lyase. The oxidative cleavage yields oxoacids, aldehydes, and allyl alcohols [15, 25].

#### 3.2.5. Ketones

2-pentanone was the major constituent among the ketones, which, altogether, accounted for the 3.97% of the identified volatile constituents. In general, ketones are less abundant in the profile of volatile compounds in fruits. 2-propanone, 2-heptanone, methyl heptenone, geranyl acetone, and *β*-ionone were the other ketones identified in Cape gooseberry. The ketones can be formed by condensation of activated fatty acids [26].

#### 3.2.6. Lactones

Four lactones identified in cape gooseberry which constituted the 2.09% of the aroma and *γ*-hexalactone was the major one followed by *δ*-octalactone. A major group of fatty acid-derived flavour compounds are lactones or alkanolides, which are organoleptically important. They have generally *γ*-(4) or *δ*-(5)-lactone structures and are linear chained, and a few are even macrocyclic. The *γ*-lactones are found primarily in plants and *δ*-lactones primarily in animal products [27]. In plants, lactones are produced in a very low amount by catabolic processes and originate from their corresponding hydroxyl carboxylic acids (4- or 5-hydroxy carboxylic acid) [17]. These compounds, particularly *γ*-lactones, are important compounds in terms of their contribution to the aroma and, in general, present fruity odour descriptors [14]. The odour of these lactones depends on the chemical structure, functional groups, and the length of side chains and due to their low odour threshold, they have a high flavour value in fruits [17]. The odour of *δ*-octalactone and *γ*-hexalactone is described as being coconut-like and fruity.

#### 3.2.7. Acids

Free acids were small components (0.90%) of the volatiles, with octanoic acid as the major one. Short- and medium-chain linear carboxylic acids are probably derived from *β*-oxidation of fatty acids. During fruit ripening, fatty acids, more precisely acyl-CoA derivatives, are metabolized to shorter-chain acyl CoAs by sequentially losing 2 carbons during each round of the *β*-oxidation [28, 29]. Aliphatic acids up to C_10_ play a significant role in flavors due to their sharp, buttery, and cheese-like odors, not only on their own, but particularly as substrates in the form of their acyl CoAs for biosynthesis of other flavors [29].

#### 3.2.8. Oxides

Finally, oxides represent 0.89% of the total volatile compounds; cis-piperitone oxide was the predominant oxide followed by cis-rose oxide. cis-Rose oxide is a major natural fragrance compound that is not only present in roses but also contributes to the aroma of other flowers, fruits (e.g., lychee), or fruit derived products (e.g., Gewürztraminer wine). The aroma of cis-rose oxide is described as floral and green [30].

## 4. Conclusions

Despite its importance, literature data about the flavour compounds of volatiles of Cape gooseberry (*Physalis peruviana* L.) is scarce. Total Cape gooseberry aroma is the result of the presence of different compounds such as alcohols, esters, terpenes, aldehydes, ketones, lactones, and oxides. Among them, esters are the most important group because they are responsible for fruity and fresh flavour. HS-SPME method followed by GC and MS detector is a good procedure for the analysis of Cape gooseberry volatile compounds.

## Figures and Tables

**Table 1:** Volatile compounds of Cape gooseberry fruit obtained by HS-SPME.

Number^a^	*I* ^ b^	Compounds	%RA^c^	Identification^d^
1	749	Acetaldehyde	0.17 ± 0.01	A, B, C
2	812	2-Propanone	0.44 ± 0.02	A, B, C
3	824	Methyl acetate	0.10 ± 0.01	A, B, C
4	871	Butanal	0.21 ± 0.01	A, B, C
5	877	2-Methylpropenal	0.06 ± 0.01	A, B
6	885	Ethyl acetate	1.52 ± 0.06	A, B, C
7	900	2-Butanone	0.03 ± 0.01	A, B, C
8	911	2-Methylbutanal	0.11 ± 0.01	A, B
9	932	Ethanol	1.09 ± 0.05	A, B, C
10	974	2-Pentanone	1.11 ± 0.06	A, B, C
11	984	Methyl butanoate	1.58 ± 0.10	A, B, C
12	1011	Isobutyl acetate	0.41 ± 0.02	A, B, C
13	1016	*α*-Pinene	0.41 ± 0.01	A, B, C
14	1035	Ethyl butanoate	6.47 ± 0.32	A, B, C
15	1038	Dimethylvinylcarbinol	2.24 ± 0.11	A, B
16	1058	Camphene	0.07 ± 0.01	A, B, C
17	1063	Isopropenyl ethyl ketone	0.02 ± 0.01	A, B
18	1069	Butyl acetate	1.61 ± 0.12	A, B, C
19	1078	Hexanal	1.26 ± 0.09	A, B, C
20	1093	3,7-Dimethyl-1-octene	0.04 ± 0.01	A, B
21	1097	Isobutyl alcohol	0.91 ± 0.03	A, B, C
22	1115	Verbenene	0.04 ± 0.01	A, B
23	1118	2-Methylbutyl acetate	2.16 ± 0.17	A, B, C
24	1123	Dehydrosabinene	0.03 ± 0.01	A, B
25	1126	*sec*-Butyl butyrate	0.02 ± 0.01	A, B, C
26	1131	Ethyl pentanoate	0.01 ± 0.01	A, B, C
27	1146	1-Butanol	2.50 ± 0.16	A, B, C
28	1156	Isobutyl butanoate	1.40 ± 0.08	A, B
29	1158	*β*-Myrcene	1.11 ± 0.09	A, B, C
30	1161	Ethyl 2-butenoate	0.03 ± 0.01	A, B
31	1172	*α*-Terpinene	0.19 ± 0.01	A, B, C
32	1179	2-Heptanone	0.98 ± 0.06	A, B, C
33	1182	Heptanal	0.06 ± 0.01	A, B, C
34	1184	Methyl hexanoate	0.07 ± 0.01	A, B, C
35	1192	Limonene	0.37 ± 0.02	A, B, C
36	1204	Eucalyptol	6.67 ± 0.57	A, B, C
37	1206	2-Methyl-1-butanol	3.10 ± 0.29	A, B, C
38	1216	Butyl butanoate	2.10 ± 0.15	A, B, C
39	1231	Ethyl hexanoate	0.60 ± 0.04	A, B, C
40	1235	6-Methyl-2-heptanone	0.13 ± 0.01	A, B
41	1241	*γ*-Terpinene	0.28 ± 0.01	A, B, C
42	1248	*β*-trans-Ocimene	0.70 ± 0.05	A, B
43	1256	Cyclooctatetraene	0.08 ± 0.01	A, B
44	1263	3-Methylbutyl butanoate	0.59 ± 0.03	A, B, C
45	1268	*ρ*-Cymene	0.62 ± 0.04	A, B, C
46	1270	Hexyl ethanoate	1.49 ± 0.11	A, B, C
47	1279	*α*-Terpinolene	2.16 ± 0.19	A, B, C
48	1287	Octanal	0.23 ± 0.01	A, B, C
49	1296	2,3-Dimethyl-1-butanol	0.02 ± 0.01	A, B
50	1312	4-Methyl-1-pentanol	0.01 ± 0.01	A, B, C
51	1317	Heptan-2-ol	1.25 ± 0.09	A, B, C
52	1324	4-Nonanone	0.40 ± 0.03	A, B, C
53	1336	Methyl heptenone	0.63 ± 0.05	A, B
54	1353	1-Hexanol	6.87 ± 0.52	A, B, C
55	1363	*cis*-3-Hexenol	0.02 ± 0.01	A, B, C
56	1367	Rosoxide	0.13 ± 0.01	A, B
57	1379	4-Octanol	0.04 ± 0.01	A, B, C
58	1384	*trans*-3-Hexenol	0.18 ± 0.01	A, B, C
59	1388	Methyl octanoate	1.90 ± 0.17	A, B, C
60	1393	Nonanal	0.61 ± 0.05	A, B, C
61	1397	2-Norbornanone	0.10 ± 0.01	A, B
62	1404	Isophorone	0.11 ± 0.01	A, B, C
63	1417	Hexyl butanoate	0.19 ± 0.01	A, B, C
64	1421	3-Methyl-1-hexanol	0.05 ± 0.01	A, B, C
65	1423	3-Ethyl-4-heptanol	0.07 ± 0.01	A, B, C
66	1434	Ethyl octanoate	4.01 ± 0.37	A, B, C
67	1438	Cymenene	0.84 ± 0.06	A, B
68	1448	3-Octenol	0.29 ± 0.01	A, B, C
69	1454	1-Heptanol	0.25 ± 0.02	A, B, C
70	1461	6-Methyl-hept-5-en-2-ol	0.19 ± 0.01	A, B, C
71	1471	Linalool oxide	0.05 ± 0.01	A, B
72	1490	Ethylhexanol	0.21 ± 0.01	A, B, C
73	1502	Decanal	0.01 ± 0.01	A, B, C
74	1517	2-Nonadecanol	0.33 ± 0.02	A, B
75	1520	Propyl octanoate	0.88 ± 0.07	A, B, C
76	1529	Benzaldehyde	2.94 ± 0.17	A, B, C
77	1543	*cis*-Piperitone oxide	0.58 ± 0.04	A, B
78	1546	Linalool	0.41 ± 0.03	A, B, C
79	1553	Isobutyl octanoate	0.95 ± 0.07	A, B, C
80	1557	1-Octanol	0.47 ± 0.03	A, B, C
81	1564	4-Isopropyl-1-methyl-2-cyclohexen-1-ol	0.03 ± 0.01	A, B
82	1575	Isopulegol	0.08 ± 0.01	A, B, C
83	1586	Fenchol	0.20 ± 0.01	A, B, C
84	1595	Methyl decanoate	1.14 ± 0.09	A, B, C
85	1605	4-Terpineol	3.27 ± 0.26	A, B, C
86	1614	Butyl octanoate	1.47 ± 0.11	A, B, C
87	1627	*β*-Cyclocitral	1.02 ± 0.09	A, B
88	1639	Ethyl decanoate	3.39 ± 0.22	A, B, C
89	1644	Butyric acid	0.12 ± 0.01	A, B, C
90	1659	Isoamyl octanoate	0.38 ± 0.02	A, B, C
91	1682	*cis*-Verbenol	0.06 ± 0.01	A, B
92	1685	*trans*-Citral	0.15 ± 0.01	A, B, C
93	1689	1,8-menthadien-4-ol	0.38 ± 0.02	A, B
94	1699	*α*-Terpineol	2.32 ± 0.15	A, B, C
95	1706	endo-Borneol	0.30 ± 0.01	A, B, C
96	1708	Butyl 3-hydroxybutanoate	0.12 ± 0.01	A, B
97	1712	*γ*-Ethylbutyrolactone	1.43 ± 0.12	A, B, C
98	1715	Verbenone	0.12 ± 0.01	A, B, C
99	1723	Propyl decanoate	0.30 ± 0.02	A, B
100	1732	Myrcenol	0.25 ± 0.01	A, B, C
101	1735	Geranaldehyde	0.23 ± 0.01	A, B, C
102	1755	Isobutyl decanoate	0.55 ± 0.03	A, B, C
103	1766	*β*-Citronellol	0.91 ± 0.05	A, B, C
104	1785	Methyl salicylate	0.10 ± 0.01	A, B, C
105	1797	Nopol	0.08 ± 0.01	A, B
106	1802	Methyl 11-cyclopentylundecanoate	0.89 ± 0.07	A, B
107	1810	Hexyl octanoate	0.06 ± 0.01	A, B, C
108	1818	Butyl decanoate	0.55 ± 0.04	A, B, C
109	1822	Phenylethyl acetate	0.02 ± 0.01	A, B, C
110	1839	*cis*-*ρ*-Mentha-1(7),8-dien-2-ol	0.18 ± 0.01	A, B
111	1844	Ethyl dodecanoate	1.33 ± 0.09	A, B, C
112	1848	Geraniol	0.12 ± 0.01	A, B, C
113	1852	*ρ*-Cymen-8-ol	1.57 ± 0.11	A, B
114	1857	Geranyl acetone	0.05 ± 0.01	A, B, C
115	1860	Hexanoic acid	0.03 ± 0.01	A, B, C
116	1878	*cis*-Myrtanol	0.07 ± 0.01	A, B
117	1883	Benzyl alcohol	2.07 ± 0.14	A, B, C
118	1919	Phenethyl alcohol	0.12 ± 0.01	A, B, C
119	1927	*δ*-Octalactone	0.59 ± 0.03	A, B, C
120	1945	*β*-Ionone	0.19 ± 0.01	A, B, C
121	1959	Isobutyl dodecanoate	0.05 ± 0.01	A, B, C
122	1969	(-)-Caryophyllene oxide	0.02 ± 0.01	A, B
123	1999	*β*-Ionone-5,6-epoxide	0.11 ± 0.01	A, B
124	2023	Butyl dodecanoate	0.08 ± 0.01	A, B, C
125	2053	Hydrocinnamic alcohol	0.06 ± 0.01	A, B
126	2074	Octanoic acid	0.54 ± 0.03	A, B, C
127	2157	*γ*-Undecalactone	0.05 ± 0.01	A, B, C
128	2180	Nonanoic acid	0.03 ± 0.01	A, B, C
129	2221	Carvacrol	0.01 ± 0.01	A, B
130	2287	Decanoic acid	0.09 ± 0.01	A, B, C
131	2309	Farnesol	0.03 ± 0.01	A, B, C
132	2354	Neric acid	0.08 ± 0.01	A, B, C
133	2367	Dihydroactinidiolide	0.03 ± 0.01	A, B

^a^Compounds listed in the order of elution from column.

^b^Retention index on TRB-Wax column.

^c^Percentage relative area (peak area of the compound relative to total peak area of identified compounds) of three replicates. Each value is expressed as mean ± SD.

^d^A: confirmed by mass spectral data fitting NIST and Wiley libraries; B: identified by retention index and compared with those reported in the literature; C: peak enrichment on coinjection with authentic reference compounds.

## References

[1] Rodrigues E, Rockenbach II, Cataneo C, Gonzaga LV, Chaves ES, Fett R (2009). Minerals and essential fatty acids of the exotic fruit *Physalis peruviana* L. *Ciencia e Tecnologia de Alimentos*.

[2] Hamdan AMA, Trinchero GD, Sozzi GO, Cerri AM, Vilella F, Fraschina AA (1999). Ripening-related changes in ethylene production, respiration rate and cell-wall enzyme activity in goldenberry (*Physalis peruviana* L.), a solanaceous species. *Postharvest Biology and Technology*.

[3] Ramadan MF, Morsel J-T (2003). Oil goldenberry (*Physalis peruviana* L.). *Journal of Agricultural and Food Chemistry*.

[4] Kataoka H, Lord HL, Pawliszyn J (2000). Applications of solid-phase microextraction in food analysis. *Journal of Chromatography A*.

[5] Verzera A, Dima G, Tripodi G, Ziino M, Lanza CM, Mazzaglia A (2011). Fast quantitative determination of aroma volatile constituents in melon fruits by headspace-solid-phase microextraction and gas chromatography-mass spectrometry. *Food Analytical Methods*.

[6] Carasek E, Pawliszyn J (2006). Screening of tropical fruit volatile compounds using solid-phase microextraction (SPME) fibers and internally cooled SPME fiber. *Journal of Agricultural and Food Chemistry*.

[7] Berger RG, Drawert F, Kollmannsberger H (1989). The flavour of cape gooseberry (*Physalis peruviana* L.). *Zeitschrift für Lebensmittel-Untersuchung und -Forschung*.

[8] Mayorga H, Knapp H, Winterhalter P, Duque C (2001). Glycosidically bound flavor compounds of cape gooseberry (*Physalis peruviana* L.). *Journal of Agricultural and Food Chemistry*.

[9] Latza S, Ganßer D, Berger RG (1996). Carbohydrate esters of cinnamic acid from fruits of *Physalis peruviana*, *Psidium guajava* and *Vaccinium vitis-idaea*. *Phytochemistry*.

[10] Pellati F, Benvenuti S, Yoshizaki F, Bertelli D, Rossi MC (2005). Headspace solid-phase microextraction-gas chromatography-mass spectrometry analysis of the volatile compounds of Evodia species fruits. *Journal of Chromatography A*.

[11] Stein SE, Linstrom PJ, Mallard WG (2011). Retention indices. *NIST Chemistry WebBook, NIST Standard Reference Database Number 69*.

[12] Pino JA, Queris O (2010). Analysis of volatile compounds of pineapple wine using solid-phase microextraction techniques. *Food Chemistry*.

[13] Bicchi C, Drigo S, Rubiolo P (2000). Influence of fibre coating in headspace solid-phase microextraction-gas chromatographic analysis of aromatic and medicinal plants. *Journal of Chromatography A*.

[14] Nunes C, Coimbra MA, Saraiva J, Rocha SM (2008). Study of the volatile components of a candied plum and estimation of their contribution to the aroma. *Food Chemistry*.

[15] Belitz HD, Grosch W, Schieberle P (2009). *Food Chemistry*.

[16] Simkin AJ, Schwartz SH, Auldridge M, Taylor MG, Klee HJ (2004). The tomato carotenoid cleavage dioxygenase 1 genes contribute to the formation of the flavor volatiles *β*-ionone, pseudoionone, and geranylacetone. *The Plant Journal*.

[17] Osorio S, Munoz C, Valpuesta V, Hui YH (2010). Physiology and biochemistry of fruit flavors. *Handbook of Fruit and Vegetable Flavors*.

[18] Schwab W, Schreier P, Kuo TM, Gardner HW (2002). Enzymatic formation of flavor volatiles from lipids. *Lipid Biotechnology*.

[19] Wyllie SG, Fellman JK (2000). Formation of volatile branched chain esters in bananas (*Musa sapientum* L.). *Journal of Agricultural and Food Chemistry*.

[20] Beekwilder J, Alvarez-Huerta M, Neef E, Verstappen FWA, Bouwmeester HJ, Aharoni A (2004). Functional characterization of enzymes forming volatile esters from strawberry and banana. *Plant Physiology*.

[21] Rodríguez-Concepción M, Boronat A (2002). Elucidation of the methylerythritol phosphate pathway for isoprenoid biosynthesis in bacteria and plastids. A metabolic milestone achieved through genomics. *Plant Physiology*.

[22] Christensen LP, Edelenbos M, Kreutzmann S, Berger RG (2007). Fruits and vegetables of moderate climate. *Flavours and Fragrances-Chemistry, Bioprocessing and Sustainability*.

[23] Maarse H (1991). *Volatile Compounds in Foods and Beverages*.

[24] Werkhoff P, Güntert M, Krammer G, Sommer H, Kaulen J (1998). Vacuum headspace method in aroma research: flavor chemistry of yellow passion fruits. *Journal of Agricultural and Food Chemistry*.

[25] Malowicki SMM, Martin R, Qian MC (2008). Volatile composition in raspberry cultivars grown in the pacific northwest determined by stir bar sorptive extraction-gas chromatography-mass spectrometry. *Journal of Agricultural and Food Chemistry*.

[26] Perestrelo R, Fernandes A, Albuquerque FF, Marques JC, Câmara JS (2006). Analytical characterization of the aroma of Tinta Negra Mole red wine: identification of the main odorants compounds. *Analytica Chimica Acta*.

[27] Baser KHC, Demirci F, Berger RG (2007). Chemistry of essential oils. *Flavours and Fragrances-Chemistry, Bioprocessing and Sustainability*.

[28] Sanz C, Olías JM, Pérez AG, Tomas-Barberan FA, Robins RJ (1997). Aroma biochemistry of fruits and vegetables. *Phytochemistry of Fruits and Vegetables*.

[29] Schwab W, Davidovich-Rikanati R, Lewinsohn E (2008). Biosynthesis of plant-derived flavor compounds. *Plant Journal*.

[30] Alsters PL, Jary W, Nardello-Rataj V, Aubry J-M (2010). Dark singlet oxygenation of *β*-citronellol: a key step in the manufacture of rose oxide. *Organic Process Research and Development*.

